# Generalized Modules for Membrane Antigens as Carrier for Polysaccharides: Impact of Sugar Length, Density, and Attachment Site on the Immune Response Elicited in Animal Models

**DOI:** 10.3389/fimmu.2021.719315

**Published:** 2021-09-14

**Authors:** Francesca Micoli, Renzo Alfini, Roberta Di Benedetto, Francesca Necchi, Fabiola Schiavo, Francesca Mancini, Martina Carducci, Davide Oldrini, Olimpia Pitirollo, Gianmarco Gasperini, Cristiana Balocchi, Nicoletta Bechi, Brunella Brunelli, Diego Piccioli, Roberto Adamo

**Affiliations:** ^1^GSK Vaccines Institute for Global Health (GVGH), Siena, Italy; ^2^GSK, Research Centre, Siena, Italy

**Keywords:** glycoconjugate, GMMA, carrier protein, polysaccharide, vaccine

## Abstract

Nanoparticle systems are being explored for the display of carbohydrate antigens, characterized by multimeric presentation of glycan epitopes and special chemico-physical properties of nano-sized particles. Among them, outer membrane vesicles (OMVs) are receiving great attention, combining antigen presentation with the immunopotentiator effect of the Toll-like receptor agonists naturally present on these systems. In this context, we are testing Generalized Modules for Membrane Antigens (GMMA), OMVs naturally released from Gram-negative bacteria mutated to increase blebbing, as carrier for polysaccharides. Here, we investigated the impact of saccharide length, density, and attachment site on the immune response elicited by GMMA in animal models, using a variety of structurally diverse polysaccharides from different pathogens (i.e., *Neisseria meningitidis* serogroup A and C, *Haemophilus influenzae* type b, and streptococcus Group A Carbohydrate and *Salmonella* Typhi Vi). Anti-polysaccharide immune response was not affected by the number of saccharides per GMMA particle. However, lower saccharide loading can better preserve the immunogenicity of GMMA as antigen. In contrast, saccharide length needs to be optimized for each specific antigen. Interestingly, GMMA conjugates induced strong functional immune response even when the polysaccharides were linked to sugars on GMMA. We also verified that GMMA conjugates elicit a T-dependent humoral immune response to polysaccharides that is strictly dependent on the nature of the polysaccharide. The results obtained are important to design novel glycoconjugate vaccines using GMMA as carrier and support the development of multicomponent glycoconjugate vaccines where GMMA can play the dual role of carrier and antigen. In addition, this work provides significant insights into the mechanism of action of glycoconjugates.

## Introduction

During the last years, nanoparticle systems have received increased interest for the display of carbohydrate antigens. Special physico-chemical properties of nano-sized particles and the presentation of multiple saccharide epitopes support the development of novel and more effective glycoconjugate vaccines (1–5). Among nanoparticles, outer membrane vesicles (OMVs) combine antigen presentation with intrinsic adjuvant properties (5–7). Traditionally, outer membrane protein complex (OMPC) from *Neisseria meningitidis* has been used as carrier for *Haemophilus influenzae* type b conjugate vaccine (8, 9). OMPC has been shown to possess TLR2-mediated adjuvant activity (10) and may contain TLR4 agonists such as lipopolysaccharides (LPS) since they derive from the outer membrane of Gram-negative bacteria.

More recently, *Escherichia coli* OMVs have been used as carriers for the display of heterologous polysaccharides (PS), resulting in glycoengineered OMVs (glyOMVs) (11). *Streptococcus pneumoniae* CPS14 capsule, for example, displayed on engineered *E. coli* OMVs induced IgG levels and efficacy in opsonophagocytic activity tests comparable with those induced by PCV13 (12).

Generalized Modules for Membrane Antigens (GMMA), OMVs naturally released from Gram-negative bacteria genetically manipulated to increase blebbing and modulate toxicity through modification of the lipid A portion of LPS (13, 14), have recently been proposed as delivery systems for O-antigen chains naturally present on their surface (15–19). O-antigens displayed on non-typhoidal *Salmonella* GMMA have been shown to induce high levels of anti-O-antigen-specific IgG antibodies, comparable with corresponding CRM_197_ conjugates formulated on alum (20). However, GMMA enhanced the IgG antibody isotype profile resulting in greater serum bactericidal activity than traditional protein conjugates. More recently, we have proposed GMMA as carrier for heterologous PS through chemical conjugation, and we have shown that GMMA glycoconjugates promote equal or enhanced saccharide immunogenicity as compared with more traditional glycoconjugates with CRM_197_ carrier protein (21).

It is well known that parameters such as saccharide length and density, conjugation chemistry, and attachment site can impact the immune response induced by glycoconjugate vaccines (22). Impact of such variables on the immune response elicited by OMV-based vaccines has not been greatly explored so far. Here, we have used different conjugation strategies to verify impact of saccharide length, density, and attachment site to proteins or LPS and lipooligosaccharide (LOS) molecules on GMMA surface on the immune response in animal models. Saccharide from different pathogens, having different structures, has been used as models and conjugated to GMMA from different pathogens.

## Materials and Methods

### Source of Generalized Modules for Membrane Antigens and Antigens

*Salmonella* Typhimurium GMMA (obtained from 1418 Δ*tolR* mutant strain) and meningococcal B (MenB) GMMA (produced from four knock-out Δ*synX*, Δ*ctra*, Δ*gna33*, and Δ*lpxL1 N. meningitidis* strains) were produced and characterized as previously described (15, 23). Meningococcal serogroup A (MenA), meningococcal serogroup C (MenC), and *H. influenzae* type b (Hib) oligosaccharides were provided by GSK. Vi and streptococcal Group A Carbohydrate (GAC) PS were purified as previously described (24–27). MenA and Vi PS of reduced length were generated as previously described (26, 28).

### Synthesis and Characterization of the Generalized Modules for Membrane Antigens Conjugates

Conjugates were synthesized as described below. The main characteristics of all the conjugates tested in this study are reported in **Table 1**.

**Table 1:** Conjugation conditions used and main characteristics of the GMMA conjugates tested in this study.

Conjugate	Chemistry	Targeting on GMMA	Saccharide length	Conjugation conditions	Antigen/GMMA w/w % ratio in purified conjugate	Number saccharide chains/GMMA particle
(1) MenA–(MenB)GMMA SIDEA	SIDEA	Proteins	1.6–3.9 kDa	GMMA/OS w/w ratio of 1:1.4; [GMMA] 11.5 mg/ml, pH 7.2, ON, RT	2.1	1,343
DP 5–12						
(2) MenA–(MenB)GMMA SIDEA	SIDEA	Proteins	5.2–8.5 kDa	GMMA/OS w/w ratio of 1:4; [GMMA] 11.5 mg/ml, pH 7.2, ON, RT	4.6	1,317
DP 16–26						
(3) MenA–(MenB)GMMA SIDEA	SIDEA	Proteins	>11.7 kDa	GMMA/OS w/w ratio of 1:4; [GMMA] 11.5 mg/ml, pH 7.2, ON, RT	4.8	726
DP > 36						
(4) MenA–(MenB)GMMAox	Reductive amination*	LOS	1.6–3.9 kDa	GMMA/OS w/w ratio of 1:10; [GMMA] 8 mg/ml, pH 7.2, ON, 30°C	2.9	2,331
DP 5–12						
(5) MenA–(MenB)GMMAox	Reductive amination*	LOS	5.2–8.5 kDa	GMMA/OS w/w ratio of 1:10; [GMMA] 8 mg/ml, pH 7.2, ON, 30°C	3.8	1,252
DP 16–26						
(6) MenA–(MenB)GMMAox	Reductive amination*	LOS	> 11.7 kDa	GMMA/OS w/w ratio of 1:10; [GMMA] 8 mg/ml, pH 7.2, ON, 30°C	4.4	763
DP > 36						
(7) Hib-(MenB)GMMA	SIDEA	Proteins	4.5 kDa	GMMA/OS w/w ratio of 1:3; [GMMA] 10.5 mg/ml, pH 7.2, ON, RT	8.1	4,230
(8) Hib-GMMAox	Reductive amination*	LOS	3.7 kDa	GMMA/OS w/w ratio of 1:6; [GMMA] 9.4 mg/ml, pH 6, ON, RT	8.4	4,414
(9) MenA–(MenB)GMMA LD	SIDEA	Proteins	4.5 kDa	GMMA/OS w/w ratio of 1:3; [GMMA] 1.7 mg/ml, pH 7.2, ON, RT	1.4	673
(10) MenA–(MenB)GMMA HD	SIDEA	Proteins	4.5 kDa	GMMA/OS w/w ratio of 1:10; [GMMA] 10 mg/ml, pH 7.2, ON, RT	5.4	2,490
(11) MenC-(MenB)GMMA LD	SIDEA	Proteins	4.5 kDa	GMMA/OS w/w ratio of 1:3; [GMMA] 1.7 mg/ml, pH 7.2, ON, RT	1.4	650
(12) MenC-(MenB)GMMA HD	SIDEA	Proteins	4.5 kDa	GMMA/OS w/w ratio of 1:10; [GMMA] 10 mg/ml, pH 7.2, ON, RT	10	4,523
(13) Vi(48.5 HD)-STm GMMA	Reductive amination*	LPS	48.5 kDa	GMMA/Vi w/w ratio of 1:3; [GMMA] 4.3 mg/ml, pH 4.5, ON, 37°C	43	93
(14) Vi(48.5 LD)-STm GMMA	Reductive amination*	LPS	48.5 kDa	GMMA/Vi w/w ratio of 1:3; [GMMA] 3.4 mg/ml, pH 7.2, ON, 37°C	8	17
(15) Vi(3.8 HD)-STm GMMA	Reductive amination*	LPS	3.8 kDa	GMMA/Vi w/w ratio of 1:1; [GMMA] 4.2 mg/ml, pH 4.5, ON, 37°C	5	138
(16) Vi(3.8 HD)-STm GMMA	Reductive amination*	LPS	3.8 kDa	GMMA/Vi w/w ratio of 1:1; [GMMA] 4.6 mg/ml, pH 6, ON, 37°C	2	55
(17) Vi-STm GMMAox	Reductive amination*	LPS	48.5 kDa	GMMA/Vi w/w ratio of 1:1; [GMMA] 2.8 mg/ml, pH 7.2, ON, 37°C	2.4	5
(18) Vi-STm GMMA	BS3	Proteins	48.5 kDa	GMMA/Vi w/w ratio of 1:10; [GMMA] 10 mg/ml, pH 7.4, ON, RT	3	7
(19) GAC-STm GMMA	Reductive amination*	LPS	7 kDa	GMMA/GAC w/w ratio of 1:1; [GMMA] 5 mg/ml, pH 4.5, ON, RT	20	1,498

ON, overnight; RT, room temperature; LPS, lipopolysaccharide, LOS, lipooligosaccharide; SIDEA linker, adipic acid bis(N-hydroxysuccinimide); BS3 linker, bissulfosuccinimidyl suberate; GMMA, Generalized Modules for Membrane Antigens; GAC, Group A Carbohydrate.

*Saccharide terminally activated with adipic acid dihydrazide (ADH).

#### Conjugation *via* Adipic Acid Bis(*N*-hydroxysuccinimide) Chemistry

MenA, MenC, or Hib oligosaccharides terminally activated with adipic acid bis(*N*-hydroxysuccinimide) (SIDEA) as previously described (29) were added to a suspension of GMMA in NaPi 50 mM pH 7.2. The mixture was stirred overnight at room temperature. Different conjugation conditions were used according to the PS linked and the GMMA used, as detailed in **Table 1**. Conjugates were purified by ultracentrifugation (110,000 rpm, 4°C, 1 h) and recovered in phosphate-buffered saline (PBS). Ultracentrifuge Thermo Scientific Sorvall MX 150+ Micro-Ultracentrifuge equipped with Thermo Scientific S110-AT rotor (K factor = 15) and 4-ml PC Thick Walled Tubes (Thermo Scientific Cat No. 45239) filled with 2 ml of solution were used.

#### Conjugation Through Reductive Amination Chemistry

*GMMA oxidation*. MenB GMMA at concentration of 8.0 mg/ml in NaPi 100 mM pH 6 were oxidized in the presence of NaIO_4_ 5 mM for 30 min in the dark at the controlled temperature of 25°C. Excess of periodate was quenched with Na_2_SO_3_ 10 mM for 15 min at room temperature before direct addition of the PS.

*S.* Typhimurium GMMA at 5 mg/ml in 100 mM of sodium acetate pH 5 was oxidized in the presence of 10 mM of NaIO_4_ for 2 h in the dark at the controlled temperature of 25°C. GMMA were purified by ultracentrifugation (110,000 rpm, 4°C, 30 min). Oxidized GMMA were resuspended in NaPi 100 mM pH 7.2. GMMA were characterized by micro BCA (>80% recovery), dynamic light scattering (confirming no aggregation), and high-performance anion-exchange chromatography with pulsed amperometric detection (HPAEC-PAD) (13% oxidation for *S.* Typhimurium GMMA and 41% for MenB GMMA).

#### Polysaccharide Derivatization With Adipic Acid Dihydrazide Linker

Vi (26), GAC PS, MenA, and Hib oligosaccharides were solubilized in 20 mM of sodium acetate buffer pH 4.5 at 30–40 mg/ml final concentration and added with ADH linker and NaBH_3_CN at a 1:1.2:1.2 w/w ratio. The solutions were mixed at 30°C for 5 days. The derivatized PS were purified by chromatography on two PD10 column equilibrated with 3 M of NaCl and then water. HPAEC-PAD was used for saccharide quantification, while TNBS colorimetric methods were used to check derivatization degree (100% for Vi, 56% for GAC, and >80% for MenA and Hib) (30). Free ADH was estimated by reversed-phase ultra-performance liquid chromatography (RP-UPLC) (<10% free NH_2_) (31).

#### Conjugations

Oxidized GMMA were added to the activated PS in the presence of NaBH_3_CN. Reaction conditions used for each conjugate are detailed in **Table 1**. The reaction was incubated overnight and purified by ultracentrifugation (110,000 rpm, 4°C, 1 h). The purified conjugate was resuspended in PBS.

#### Conjugation *via* Bissulfosuccinimidyl Suberate Chemistry

*S*. Typhimurium GMMA, at a protein concentration of 4.0 mg/ml in MES buffer pH 6, was added to BS3 linker at a final concentration of 50 mg/ml in the reaction mixture. The mixture was incubated at 25°C for 30 min; then activated GMMA were purified by ultracentrifugation (110,000 rpm, 16 min, 4°C). Resulting GMMA (70% recovery by micro BCA) had 43.8% of NH_2_ groups derivatized with the BS3 linker according to TNBS colorimetric method (30).

After GMMA-BS3 ultracentrifugation, Vi-ADH was immediately added. In the conjugation step, a 1:10 w/w ratio of GMMA to Vi-ADH was used, at Vi-ADH concentration of 100 mg/ml in NaPi 50 mM pH 7. After overnight incubation at room temperature (RT), the conjugate was purified by ultracentrifugation (110,000 rpm, 1 h, 4°) and recovered in PBS.

### Conjugate Characterization

Conjugates were characterized by micro BCA for total protein recovery (21), while amount of saccharide antigen linked was determined by HPAEC-PAD after performing acid hydrolysis directly on GMMA as previously described (24, 32–36). It was verified that there was no interference from GMMA in the quantification of each saccharide. A known amount of the conjugated PS was physically mixed to GMMA, and it was verified that analysis by HPAEC-PAD gave results comparable with those obtained by testing the same amount of the PS alone. For MenA, MenC, Hib, and GAC saccharides, conjugate formation was also confirmed by sodium dodecyl sulfate–polyacrylamide gel electrophoresis (SDS-PAGE)/Western blotting as previously described (21). For MenA, MenC, and GAC conjugates, polyclonal sera internally generated in mice were used as primary antibodies, while for Hib conjugate, a commercial antibody (Bacto Hib DIFCO 2236-50-1) was used. NanoTracking Analysis (NTA) was used to count the number of GMMA particles in solution and estimate the number of PS chains per GMMA. NS300 Nanosight instrument (Malvern) equipped with a CMOS camera and a 488-nm monochromatic laser beam was used. Data acquisition and processing were performed using NTA software 3.2 build 3.2.16, and more details on the analysis can be found in De Benedetto et al. (23).

Percentage of free saccharide was calculated by solid-phase extraction (SPE) using a C4 cartridge (Vydac BioSelect) followed by HPAEC-PAD analysis for MenA, MenC, and Hib (33, 35), by high-performance liquid chromatography–size-exclusion chromatography (HPLC-SEC) (refractive index detection) for Vi and GAC conjugates.

### Immunogenicity Studies in Animal Models

All animal sera used in this study were derived from mouse or rat immunization experiments performed at the GSK Animal Facility in Siena or at Toscana Life Sciences Animal Facility (Siena, Italy), in compliance with the relevant guidelines (Italian D. Lgs. n. 26/14 and European directive 2010/63/UE) and the institutional policies of GSK. The animal protocols were approved by the Animal Welfare Body of GSK, Siena, Italy, and by the Italian Ministry of Health (Approval number 804/2015-PR) and Animal Welfare Body of Toscana Life Sciences and by the Italian Ministry of Health (Approval number 479/2017-PR).

CD1 5-week-old female mice were immunized subcutaneously (s.c) or intramuscularly (i.m.) at days 0 and 28. CD1 5-week-old female nude mice (devoid of mature T cells) were immunized s.c. at days 0 and 28 (37). Crl : CD 8-week-old female rats were immunized i.m. at days 0 and 28. Mice and rats were bled from the retromandibular plexus and the tail vein, respectively. Rats were preheated for 5 min in a warming cage at 37°C before bleeding. Final bleed in rats was performed under general anesthesia (alfaxalone 20 mg/kg + medetomidine 0.05 mg/kg + fentanyl 0.1 mg/kg). Blood was kept at 37°C up to 2 h or at RT up to 3 h in the untreated collection tubes and then centrifuged for 10 min at 2,851 rcf, 4°C before serum collection. Animal models, immunization routes, and schemes were selected according to the PS antigens tested. Anti-antigen-specific IgG levels were measured at days −1, 27, and 42 (40 for the study in rats) by enzyme-linked immunosorbent assay (ELISA) (38). Purified O-antigen from S. Typhimurium and Streptococcal Group A Carbohydrate conjugated to human serum albumin (HSA) were used for ELISA plate coating at 5 and 1 µg/ml, respectively, in carbonate buffer pH 9.6; purified Vi at 1 µg/ml in phosphate buffer pH 7.0; purified MenA and MenC capsular PS were used at 5 µg/ml in PBS pH 8.2; and Hib PS conjugated to HSA was used at 2 µg/ml in PBS pH 7.4. ELISA units were expressed relative to a mouse antigen-specific antibody standard serum curve, with the best five-parameter fit determined by a modified Hill plot. One ELISA unit is defined as the reciprocal of the dilution of the standard serum that gives an absorbance value equal to 1 in this assay. Each mouse serum was run in triplicate.

Serum bactericidal antibody (SBA) against meningococcal (MenA, MenC, and MenB) strains was tested using baby rabbit complement as previously described (29, 39). F8238 MenA, C11 MenC, NZ98/254, M08-0240104, and M01-0240320 MenB strains were used. Pooled sera from each group were tested by SBA.

### Statistical Analysis

Datasets were analyzed using two-tailed non-parametric Mann–Whitney test (for comparing the same time point for two different groups) or one-tailed non-parametric Wilcoxon matched-pairs signed rank test (for comparing different time points for a same group) with Prism (GraphPad Software). p-Values less than 0.05 were considered statistically significant.

## Results

### Chemical Linkage of Polysaccharides to Generalized Modules for Membrane Antigens

**Figure 1:**
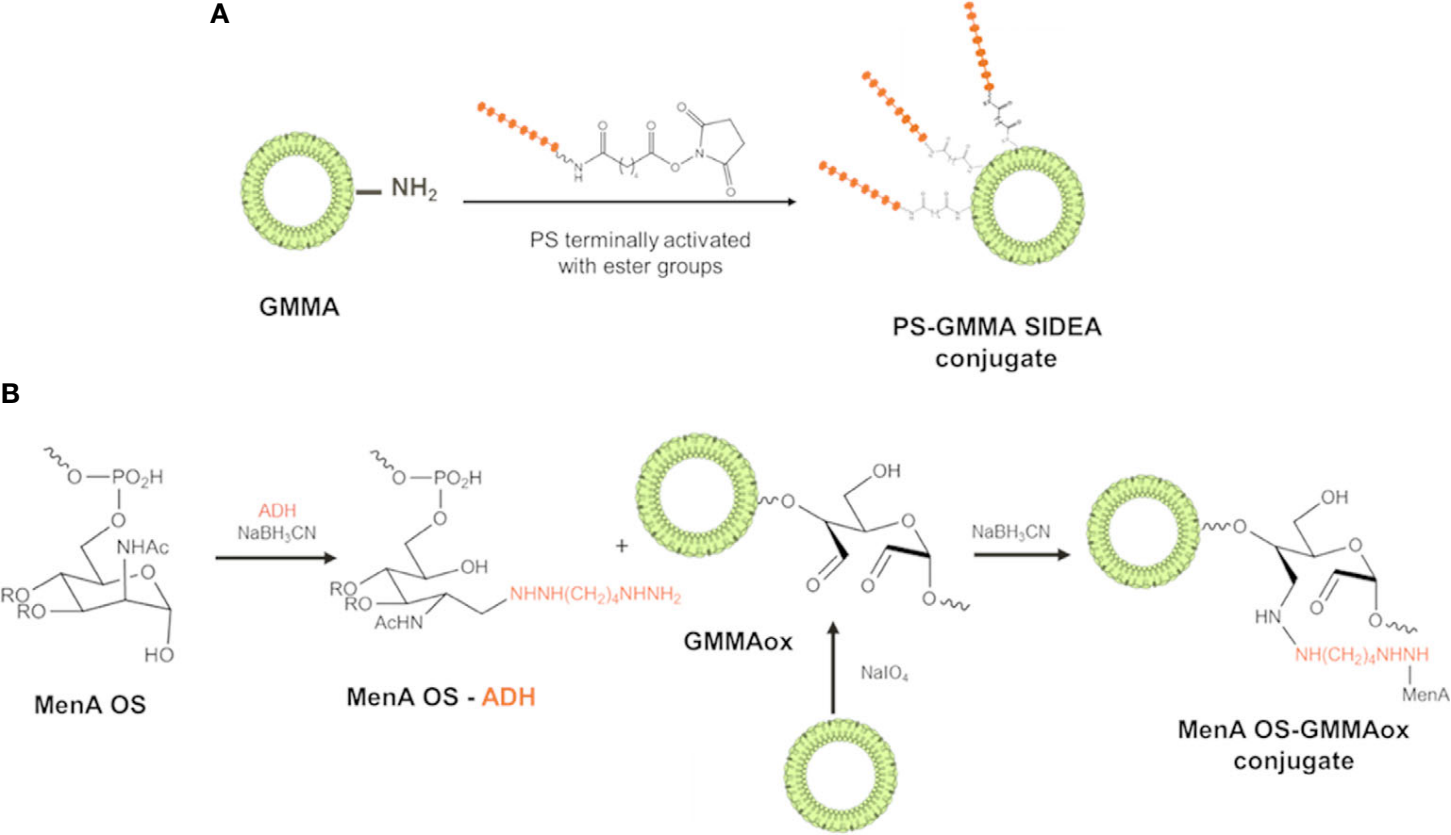
Conjugation schemes used for linkage of PS to GMMA. **(A)** PS were terminally activated with adipic acid bis(*N*-hydroxysuccinimide) (SIDEA) linker and randomly conjugated to lysines of GMMA surface proteins. **(B)** PS (MenA structure reported as example) were terminally derivatized with adipic acid dihydrazide (ADH) and linked to LPS/LOS on oxidized GMMA by reductive amination. PS, polysaccharides; GMMA, Generalized Modules for Membrane Antigens; ADH, adipic acid dihydrazide; LPS, lipopolysaccharides; LOS, lipooligosaccharides.

PS with different structural features and size were conjugated to GMMA from different pathogens. MenA, MenC, and Hib oligosaccharides were terminally activated with SIDEA linker (29) and randomly conjugated to lysines of GMMA surface proteins (**Figure 1A**). Alternatively, the oligosaccharides were terminally derivatized with ADH and linked to LOS on oxidized GMMA by reductive amination (**Figure 1B**).

**Figure 2:**
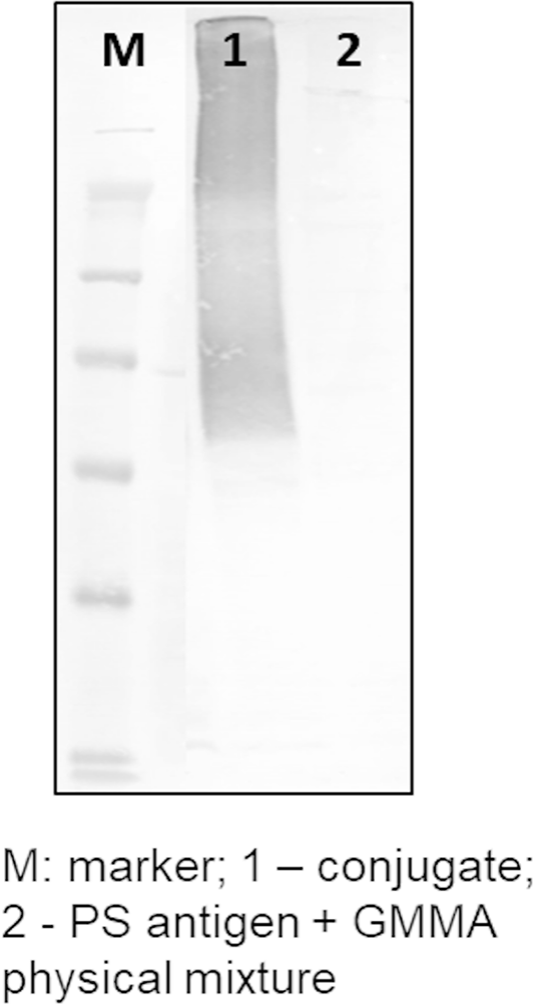
Conjugate formation proved by Western blotting analysis.

A similar approach was used for linkage of streptococcal GAC and *S*. Typhi Vi PS to GMMA. By playing with conjugation conditions, in particular by using different saccharide-to-protein molar ratios (as for meningococcal oligosaccharides) or different buffer pH (as for Vi), it was easy to modulate the number of sugar chains per GMMA particle (**Table 1**). Formation of saccharide–GMMA conjugates was verified by Western blotting analysis (**Figure 2**); and the amount of total saccharide and total protein were quantified by HPAEC-PAD and micro BCA, respectively. The saccharide-to-protein w/w ratio, coupled with an estimate of the number of GMMA particles per ml measured by NTA, allowed us to count the average number of saccharide chains per GMMA particle (**Table 1**).

### Impact of Saccharide Length, Density, and Attachment Site on the Immune Response Induced by Generalized Modules for Membrane Antigens Conjugates

**Figure 3:**
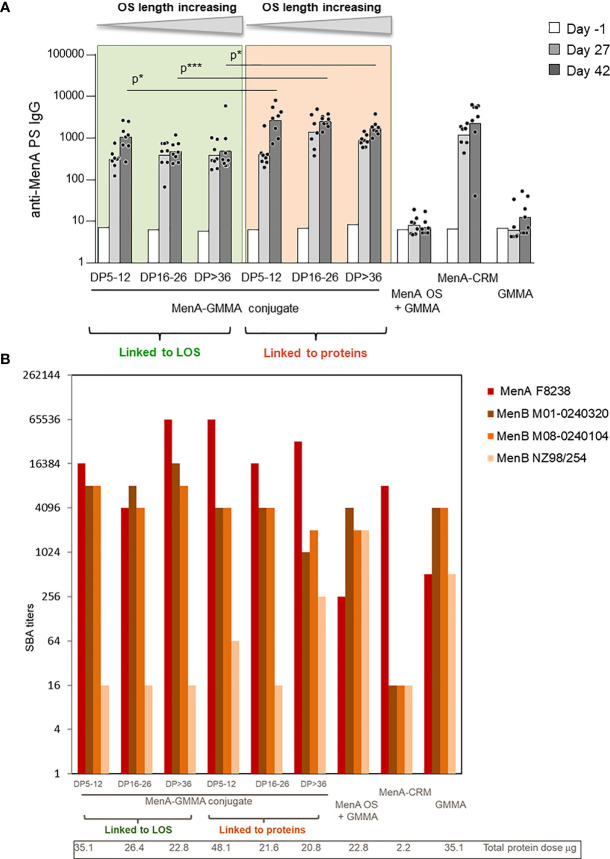
Impact of saccharide length and attachment site on the immune response induced by MenA–MenB GMMA conjugates. Eight CD1 mice per group were immunized i.m. at days 0 and 28, with 1 μg MenA/dose in the presence of Alhydrogel. Sera were collected at days −1, 27, and 42. **(A)** Summary graphs of anti-MenA PS IgG geometric mean units (bars) and individual antibody levels (dots) are reported **(A)**. SBA titers of pooled sera collected 2 weeks after second injection against MenA and MenB strains are reported **(B)**. GMMA, Generalized Modules for Membrane Antigens; PS, polysaccharides; SBA, serum bactericidal antibody. * 0.01 < p < 0.5; *** 0.0001 < p < 0.001.

The impact that sugar length and linkage site on GMMA could have on the immune response induced by the conjugates was initially studied with MenA oligosaccharides linked to MenB GMMA. MenA oligosaccharides of different and non-overlapping length (polymerization degree (DP) equal to 5–12, 16–26, and >36) were conjugated to proteins or LOS on MenB GMMA (**Table 1**, constructs 1–6) and tested in mice. GMMA alone or physically mixed to MenA oligosaccharides were used as negative controls, while MenA–CRM_197_ conjugate was the positive control. Conjugation to proteins or LOS on GMMA resulted in induction of a strong anti-MenA IgG response at a level comparable with that of MenA–CRM_197_. We found that the sugar length did not influence MenA-specific serum IgG response, because no difference in antibody production was observed after immunization with the different MenA–GMMA conjugates (**Figure 3A**), regardless of whether conjugation was directed to LOS or proteins. Interestingly, the conjugates generated from saccharides attached to proteins invariably elicited a higher MenA-specific IgG response 2 weeks after second immunization compared with MenA oligosaccharides linked to LOS (**Figure 3A**). However, all GMMA conjugates, independently from the attachment site of meningococcal oligosaccharides to GMMA, induced antibodies with bactericidal activity against a homologous MenA strain (**Figure 3B**).

The ability of MenB GMMA to induce an immune response after attachment of MenA oligosaccharides was verified by testing the bactericidal activity of antibodies induced against three different MenB strains (**Figure 3B**). While bactericidal activity against UK320 and UK104 strains was not impaired by conjugation, the one against the New Zealand strain was negatively impacted. As bactericidal activity against this strain is mainly mediated by the PorA antigen on MenB GMMA (40, 41), we could speculate that the random conjugation of MenA oligosaccharides to proteins on GMMA could impact on PorA structure and conformation. However, the same was true for the glycoconjugates obtained by linkage of oligosaccharides to LOS on MenB GMMA, indicating that probably the saccharide chains masked some protein components shifting the immune response toward themselves.

**Figure 4:**
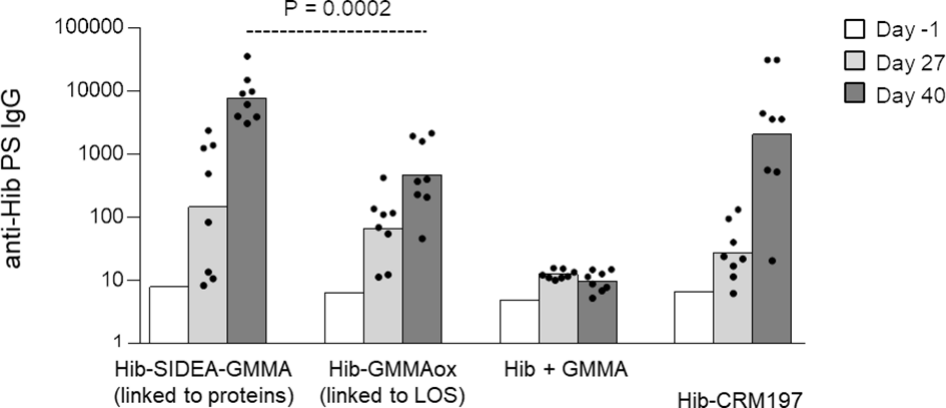
Hib oligosaccharides were conjugated to proteins (Hib-SIDEA-GMMA) or LOS (Hib-GMMAox) of GMMA. Resulting conjugates were compared in rats together with Hib physically mixed to GMMA and Hib-CRM_197_ with Alhydrogel. Eight adult rats per group were i.m. immunized at days 0 and 28 (0.5 μg Hib/dose). Sera were collected at days −1, 27, and 40 and analyzed for anti-Hib PS IgG response. Summary graphs of anti-PS IgG geometric mean units (bars) and individual antibody levels (dots) are reported. LOS, lipooligosaccharides; GMMA, Generalized Modules for Membrane Antigens.

Next, Hib oligosaccharides were conjugated to MenB GMMA by targeting proteins or LOS (constructs 7–8, **Table 1**), and the Hib-specific serum IgG response was measured in rats, in comparison with Hib oligosaccharides mixed to GMMA and Hib-CRM_197_ conjugate (**Figure 4**). As observed for MenA conjugates, also in this case, the conjugate obtained by linkage of Hib to proteins induced a stronger anti-Hib IgG response than the conjugate produced by linking oligosaccharides to LOS. Both GMMA conjugates elicited anti-Hib PS IgG titers significantly higher than Hib simply mixed to GMMA and comparable with Hib-CRM_197_.

**Figure 5:**
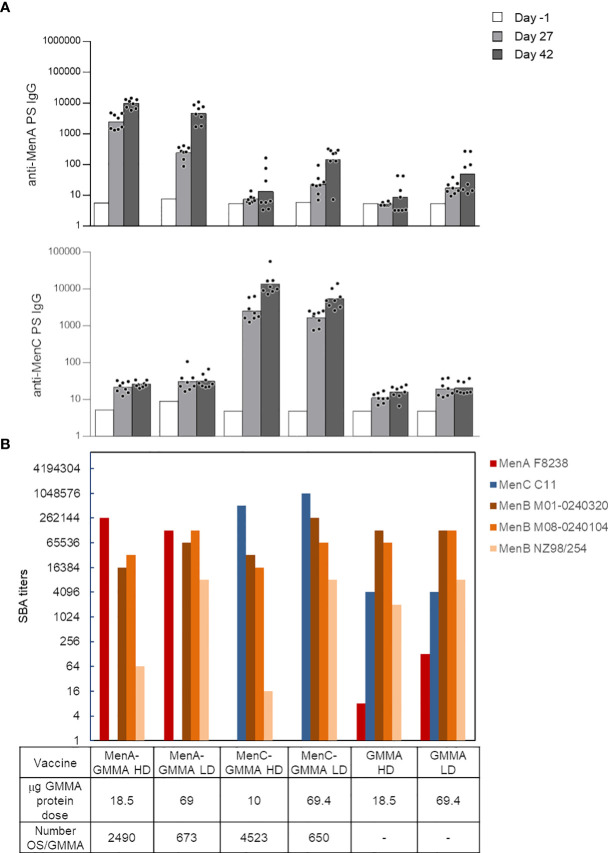
Impact of saccharide density on the immune response induced by MenA/MenC–MenB GMMA conjugates. Eight CD1 mice per group were immunized i.m. at days 0 and 28, with 1 μg MenA/MenC/dose in the presence of Alhydrogel. Sera were collected at days −1, 27, and 42. Anti-MenA and MenC IgG response **(A)** and SBA titers of pooled sera collected 2 weeks after second injection against MenA, MenC, and MenB strains **(B)** are reported. HD, high density; LD, low density. Summary graphs of IgG geometric mean units (bars) and individual antibody levels (dots) are reported. In **(B)**, absent bars for MenA and MenC strains represent measures not done. GMMA alone were used as control. GMMA, Generalized Modules for Membrane Antigens; SBA, serum bactericidal antibody.

After having investigated the impact of saccharide length and attachment *via* proteins or LOS on GMMA, we interrogated the effect of the density of saccharide conjugated to GMMA particles, which is another important feature for glycoconjugate vaccines. We produced conjugates differing for the average number of MenA or MenC oligosaccharides linked per GMMA particle (constructs 9–12, **Table 1**). No major impact of oligosaccharide density on anti-PS IgG response (**Figure 5A**) and functionality of the sera induced in mice was found (**Figure 5B**). However, control of glycosylation density could be useful to fully preserve the immune response induced by GMMA per se. In fact, by testing the bactericidal activity of the sera against a panel of different MenB strains, we assessed that the largest was the number of meningococcal oligosaccharide chains conjugated per GMMA particle, and the highest was the impact on the functionality of the elicited sera (this was particularly evident for MenB NZ98/254 strain). Therefore, linkage of fewer sugar chains per GMMA particle seems preferable.

**Figure 6:**
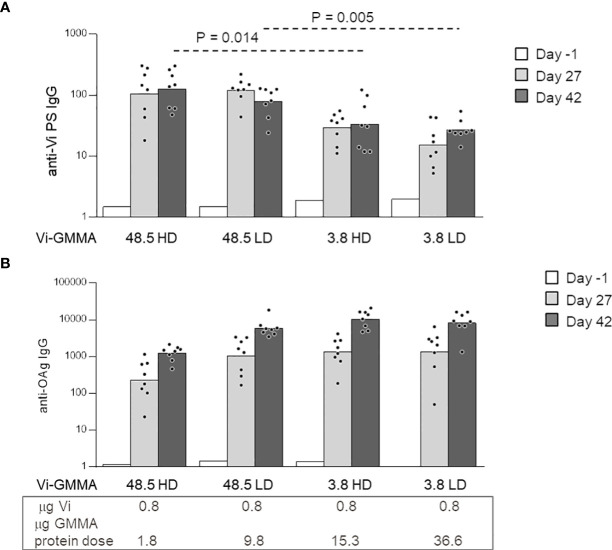
Impact of saccharide length and density on the immune response induced by Vi-*Salmonella* Typhimurium GMMA conjugates. Eight CD1 mice per group were immunized s.c. at days 0 and 28, with 0.8 μg Vi/dose with no Alhydrogel. Sera were collected at days −1, 27, and 42 and analyzed for anti-Vi **(A)** and anti-*S.* Typhimurium O-antigen (OAg) **(B)** IgG response. Summary graphs of IgG geometric mean units (bars) and individual antibody levels (dots) are reported. HD, high density; LD, low density; GMMA, Generalized Modules for Membrane Antigens.

To further explore the effect of glycan length and density with a larger-size PS, conjugates formed by *S*. Typhi Vi PS attached to *S.* Typhimurium GMMA were generated (constructs 13–16, **Table 1**). Linkage of Vi to LPS on GMMA allowed to introduce a different number of PS chains to GMMA, while conjugation to proteins resulted in few Vi chains per GMMA particle only. As previously verified for MenA and MenC (**Figure 5**), we did not find impact of antigen density on Vi-specific serum IgG response. However, the saccharide length in this case generated a significant effect, because longer Vi PS (48.5 kDa) were able to induce significantly higher Vi-specific serum IgG titers than the shorter Vi (3.8 kDa) (**Figure 6A**). All conjugates induced anti-*S.* Typhimurium O-antigen IgG response, preserving immunogenicity of GMMA per se (**Figure 6B**).

### Glycoconjugation to Generalized Modules for Membrane Antigens Promotes a Shift Toward a T-Independent Humoral Immune Response Based on the Type of Conjugated Saccharide

The display of PS on GMMA, especially if in high-density modality, generates repetitive epitope moieties on GMMA surface that can facilitate cognate B-cell receptor cross-linking, which could lead to B-cell activation in the absence of T-cell help. This shift toward a strong and fast T-independent B-cell stimulation would not promote germinal center formation and the consequent generation of long-lived plasma cells secreting high-affinity antibodies and memory B cells. Therefore, the T-independent B-cell response can have a negative impact on the efficacy of the humoral immune response, especially in infants or young children, and a detrimental effect on immunological memory and persistence of the antibody response. To investigate any potential T-independent nature of the humoral immune response induced by PS conjugated on GMMA, we evaluated different PS–GMMA conjugates immunizing wild-type and nude mice, devoid of mature T cells. We used MenC, Vi, and GAC PS conjugated to *S*. Typhimurium GMMA, so as to test PS with different structural features (constructs 12 and 17–19, **Table 1**).

**Figure 7:**
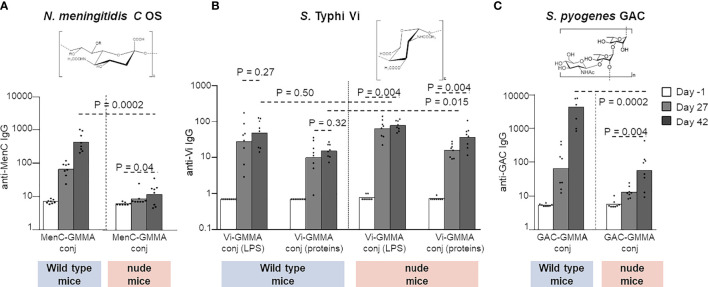
Different PS were linked to GMMA and tested in wild-type or CD1 nude mice. Eight mice per group were s.c. immunized at days 0 and 28 with 1 μg MenC oligosaccharide **(A)**, 0.8 μg Vi PS **(B)**, or 1 μg GAC **(C)**. All formulations were tested in the absence of Alhydrogel. Sera were collected at days −1, 27, and 42 and analyzed for anti-PS-specific IgG response. Summary graphs of IgG geometric mean units (bars) and individual antibody levels (dots) are reported. PS, polysaccharides; GMMA, Generalized Modules for Membrane Antigens; GAC, Group A Carbohydrate.

MenC–GMMA conjugate was unable to induce a significant MenC-specific antibody response in nude mice compared with wild-type animals, clearly confirming the need of the T-cell help to promote a humoral response against MenC oligosaccharide (**Figure 7A**). On the contrary, Vi-GMMA induced a strong anti-Vi-specific serum IgG response in wild-type as well as nude mice, revealing that this PS promoted a T-independent humoral immune response (**Figure 7B**). Antibody levels induced in wild-type mice were high after the first dose, with no booster after re-injection. The same was verified by linking Vi PS to LPS or proteins on GMMA. Using GAC–GMMA conjugate, we observed an intermediate situation, since nude mice immunized with GAC–GMMA conjugate generated a GAC-specific serum IgG response, which was significantly lower than that induced in wild-type mice (**Figure 7C**). Thus, GMMA-conjugated GAC PS elicit a weak T-independent saccharide response.

## Discussion

Conjugation to appropriate carrier proteins, providing T-cell help, is an established way for improving immunogenicity of PS antigens giving rise to immunological memory, isotype switching, affinity maturation, persistence of antibody response, and ability to induce adequate protection in infants and children under 2 years of age (42–46). Few carrier proteins have been used so far for licensed glycoconjugates (1), highlighting in certain cases reduced immunogenicity against the PS hapten due to preexisting immunity toward the protein (the so-called “carrier epitope suppression”) (47). Recent years have seen efforts to identify alternative carrier proteins, particularly with a dual role of carrier and antigen (3).

Recently, we have proposed GMMA as carrier for heterologous PS, showing the ability to enhance the antigen-specific humoral immune response compared with the antigen alone or physically mixed with GMMA (21).

Compared with traditional carrier proteins, GMMA are nanoparticle systems, with optimal size for immune stimulation and presenting multiple copies of the PS favoring B-cell activation. They possess immunostimulatory molecules, such as LPS, lipoproteins, or peptidoglycans, that can stimulate innate immunity and consequently enhance adaptive immunity (5, 7). Importantly, GMMA can be produced using a simple manufacturing process (15, 48). Here, we developed conjugation chemistries to easily and efficiently link PS differing in structure and size to GMMA surface, targeting both LPS and proteins on GMMA. Also, conjugation to GMMA was modulated not to impact on the immune response induced by GMMA as antigen. This supports the use of GMMA with a dual role of carrier and antigen for the development of multicomponent vaccines covering various diseases at the same time. In particular, *Salmonella* and meningococcal diseases, here used as models, are both common in several countries of sub-Saharan Africa (49–51); Hib and MenB are two critical etiological agents of meningitis, and a unique pan-meningococcal vaccine could offer a unique opportunity to combat the meningococcal meningitis worldwide; finally, *S.* Typhi and non-typhoidal *Salmonella* are leading causes of disease and mortality in Africa (52).

Antigen length and density are parameters that can play a role on the immune response elicited by glycoconjugate vaccines (22). Here, by playing with these features on GMMA, we observed that PS density seems not to play major role on the anti-saccharide-specific immune response induced in mice. Also, a limited number of oligosaccharide chains linked to GMMA is sufficient for inducing a strong immune response. On the other hand, saccharide length can play a role depending on the specific PS used. This confirms that, similar to traditional glycan–protein conjugates, saccharide length needs to be investigated and optimized specifically for each antigen of interest.

Another relevant aspect of this work is the observation that the carrier effect of GMMA for PS is observed irrespectively of whether the antigen is linked on GMMA proteins or LPS/LOS and can be dependent or not on T-cell help, based on the nature of the PS. Recently, it has been shown that glycan–protein conjugates induce a T-cell-dependent response through generation in B cells of peptides or glycopeptides (depending on the nature of the conjugated sugar) that are presented to the helper T cells (53, 54). Our finding suggests that the immunological mechanisms of the “carrier” effect of GMMA for PS could be the result of different coexisting mechanisms, which would be also depending on the nature of the PS linked.

Interestingly, even when GAC was linked to LPS on GMMA, the immune response was strongly mediated by T-cell activation as verified by the much lower response induced by the conjugate in nude mice. This supports the finding that direct linkage of PS to proteins is not needed, although co-presentation seems crucial. It is important that the interaction between protein and PS moieties is strong enough to allow internalization in the same B cell to assure T-cell engagement (3). Finally, our data show that linkage of certain PS (e.g., MenA and Hib) to proteins on GMMA can result in higher anti-PS-specific IgG response and seems preferable to conjugation to the LPS. Recently, *E. coli* glycoengineered OMVs have been proposed for the expression of heterologous PS that are anchored to lipid A-core as acceptor (11, 12, 55). Our findings can be informative also for this approach. Compared with the chemical conjugation proposed here, glycoengineering holds the potential for simplified and lower-cost vaccine production. However, chemical conjugation can be more easily applied to OMVs/GMMA from different pathogens and to PS with different structures and can represent a fast tool to investigate how parameters such as those investigated here impact the immune response elicited by these novel glycoconjugates, including glycoengineered OMVs.

Also additional nanoparticle systems, such as Qβ (56, 57) and hepatitis B core antigen virus-like particles (58), are being proposed as novel carrier systems to provide a strong anti-PS immune response. It will be interesting to compare GMMA and OMVs with these other systems for their ability to induce strong response after one only injection, persistency of the response, memory, and ultimately efficacy in infants and to see if they will behave similarly mainly due to their particulate nature and display of multiple antigens or if there will be specific features for a difference.

In conclusion, we found that optimization of parameters such as sugar length and density is crucial to fully exploit the potential of GMMA as platform for multicomponent vaccines, where GMMA can act as T-cell helper and antigen. The action of GMMA as carrier seems independent of the direct linkage of the sugar to the protein and present some specificity that deserves to be further investigated. Additional studies, including evaluation of IgG subclasses, IgM, antibody affinity, and cellular response, will be needed to further characterize the quality of the immune response elicited by GMMA conjugates and to better understand the mechanism of action elicited by these novel carrier systems. Unraveling these immunological mechanisms could guide the design of even more effective GMMA-based vaccines and would be informative for other nanoparticle based conjugates under development.

## Data Availability Statement

The raw data supporting the conclusions of this article will be made available by the authors, without undue reservation.

## Ethics Statement

The animal study was reviewed and approved by Animal Welfare Body of GSK, Siena, Italy, and by the Italian Ministry of Health (Approval number 804/2015-PR) and Animal Welfare Body of Toscana Life Sciences and by the Italian Ministry of Health (Approval number 479/2017-PR).

## Author Contributions

FMi, RAd, and DP designed the study. FMi wrote the manuscript. FMi, RAl, RD, FS, FM, MC, DO, OP, CB, NB, GG, and BB performed the experiments and analyzed the data. FMi, FN, CB, BB, DP, and RAd supervised research and reviewed the data. All authors contributed to the article and approved the submitted version.

## Funding

The authors declare that this study received funding from GlaxoSmithKline Biologicals SA. The funder was not involved in the study design, collection, analysis, interpretation of data, the writing of this article or the decision to submit it for publication.

## Conflict of Interest

All the authors were employees of the GSK group of companies when the study was performed. FMi, RAl, and RD are listed as inventors on patents related to this work owned by the GSK group of companies. GSK Vaccines Institute for Global Health Srl is an affiliate of GlaxoSmithKline Biologicals SA.

## Publisher’s Note

All claims expressed in this article are solely those of the authors and do not necessarily represent those of their affiliated organizations, or those of the publisher, the editors and the reviewers. Any product that may be evaluated in this article, or claim that may be made by its manufacturer, is not guaranteed or endorsed by the publisher.
